# Combined Audit for depot/LAI (Long-Acting Injectable) Antipsychotic Medications Relapse Prevention and Side Effects for Older Adult Populations (Pan Trust Audit – Black Country Healthcare NHS Foundation Trust)

**DOI:** 10.1192/bjo.2025.10562

**Published:** 2025-06-20

**Authors:** Sui Yung Chen, Aparna Prasanna, Emily Cross, Kiran Bhandal, Michael Adesina

**Affiliations:** 1 Black Country Healthcare NHS Foundation Trust, Wolverhampton, United Kingdom; 2 Black Country Healthcare NHS Foundation Trust, Walsall, United Kingdom

## Abstract

**Aims:** This is an audit that combines the audit on relapse prevention and the audit on the side effects of the depot/LAI on cycle 2 with the aim of benchmarking the compliance of the clinicians’ current clinical practice compared with the POMH-UK and NICE CG178.

**Methods:** The audit was completed with a retrospective electronic medical note review between Oct 2023 and Oct 2024, including patients who were above 65 and currently on depot/LAI. Total of 61 patients were identified, but only 59 patients were included (1 deceased and 1 duplication excluded). The audit was completed using pre-designed questionnaires based on POMH-UK (relapse prevention) and NICE CG 178 (side effect).

**Results:** For relapse prevention, most of the data from cycle 2 showed improvement compared with cycle 1, where the care plan included a crisis plan (89.7%); a plan to respond when defaulting from treatment (34.3%); the review of the therapeutic responses of the depot/LAI (90.7%); and involving patients in generating their own care plan (89.7%).

There were decrease in the percentage of accessibility of the care plan in the clinical record (98.3%), documentation of the relapse “signature” signs and symptoms (39.7%), and the annual review by the prescribing team (72.9%). The lack of documentation might be contributing to the low percentage for the review of relapse “signature” signs and symptoms.

Cycle 2 is a pan trust audit, which might have impacted the decrease in the percentage of the above questions, whereas the improvement in the small percentage also reflected the significant improvement of the clinical practice since cycle 1.

For the depot/LAI medications side effects assessment, there were minimal increases in the assessment of side effects over the last 12 months (76.2%). However, there was a decrease in the percentage where the side effects were identified and followed by the change of plan (90%). The one patient who did not have the changed plan was struggling with the side effects of sedation. The common side effect identified from this audit was EPSE (extrapyramidal side effect).

**Conclusion:** Although we are not achieving 100% on each component, there is evidence of improvement in good clinical practice as shown in the result compared with cycle 1.

There is still work that needs to be done in order to address the improvement of both completing and documenting the annual review of the care plan and side effects of the patients who are on depot/LAI antipsychotic medications.

